# Lamin A/C and PI(4,5)P2—A Novel Complex in the Cell Nucleus

**DOI:** 10.3390/cells13050399

**Published:** 2024-02-25

**Authors:** Sara Escudeiro-Lopes, Vlada V. Filimonenko, Lenka Jarolimová, Pavel Hozák

**Affiliations:** 1 Department of Biology of the Cell Nucleus, Institute of Molecular Genetics of the Czech Academy of Sciences, Vídeňská 1083, 142 20 Prague, Czech Republic; sara.escudeiro_lopes@uochb.cas.cz (S.E.-L.); vlada.filimonenko@img.cas.cz (V.V.F.);; 2 Electron Microscopy Core Facility, Institute of Molecular Genetics of the Czech Academy of Sciences, Vídeňská 1083, 142 20 Prague, Czech Republic

**Keywords:** lamin A/C, phosphorylation, cell nucleus, nuclear lamina, nucleoplasm, phosphoinositides, PI(4,5)P2, nuclear myosin 1, NM1

## Abstract

Lamins, the nuclear intermediate filaments, are important regulators of nuclear structural integrity as well as nuclear functional processes such as DNA transcription, replication and repair, and epigenetic regulations. A portion of phosphorylated lamin A/C localizes to the nuclear interior in interphase, forming a lamin A/C pool with specific properties and distinct functions. Nucleoplasmic lamin A/C molecular functions are mainly dependent on its binding partners; therefore, revealing new interactions could give us new clues on the lamin A/C mechanism of action. In the present study, we show that lamin A/C interacts with nuclear phosphoinositides (PIPs), and with nuclear myosin I (NM1). Both NM1 and nuclear PIPs have been previously reported as important regulators of gene expression and DNA damage/repair. Furthermore, phosphorylated lamin A/C forms a complex with NM1 in a phosphatidylinositol-4,5-bisphosphate (PI(4,5)P2)-dependent manner in the nuclear interior. Taken together, our study reveals a previously unidentified interaction between phosphorylated lamin A/C, NM1, and PI(4,5)P2 and suggests new possible ways of nucleoplasmic lamin A/C regulation, function, and importance for the formation of functional nuclear microdomains.

## 1. Introduction

Lamins are type V intermediate filament (IF) proteins, which are the major component of the nuclear lamina, a filamentous meshwork located underneath the inner nuclear membrane [1,2]. Lamins in the nuclear lamina not only play a crucial structural and mechanical role in maintaining the nuclear integrity, but they also perform, together with their binding partners, a variety of regulatory cellular functions [2], including chromatin organization [3], transcriptional regulation [4], cell cycle regulation, and DNA damage response [5,6]. Mammals have three lamin genes: *LMNA*, *LMNB1*, and *LMNB2*. The *LMNA* gene contains 12 exons that by alternative splicing give rise to lamin C and prelamin A—a precursor or the mature lamin A, they are commonly referred as lamin A/C, or A-type lamins. *LMNB1* and *LMNB2* genes encode for lamin B1 and lamin B2, respectively [7]. All lamins display an IF domain organization: N-terminal head, rod domain, and a C-terminal tail containing the IgG fold domain, the nuclear localization signaling (NLS), and a Caax motif (for B-type lamins and prelamin A) [8]. Mutations in the *LMNA* gene cause severe diseases, named laminopathies [9], including muscular and/or skeletal dystrophies [10,11], neuropathies, lipodystrophies, and progeroid syndromes, such as Hutchinson–Gilford progeria syndrome [12,13]. Whereas several therapeutic approaches have been examined using patient cells or mouse models [14], the molecular mechanisms underlying laminopathies still remain elusive, as well as detailed mechanisms through which lamins exert their biological functions. 

In addition to the presence in the nuclear lamina, where lamins form 3.5 nm thick filaments [15], lamins can also be found at the nuclear interior, where they were first observed using electron microscopy [16]. More studies have followed showing the presence of lamins in the nucleoplasm, either in the form of intranuclear lamin foci or short undefined fibrous structures [4]. Nucleoplasmic lamin was reported to be more mobile [17], and easily extracted with detergents, suggesting a less complex assembly—soluble and unpolymerized—than the filamentous lamin in the nuclear lamina [18,19]. The molecular mechanisms that regulate the nucleoplasmic A-type lamins are still poorly understood; however, lamin phosphorylation appears to be one of important factors. Lamins undergo several types of post-translational modifications, phosphorylation being the most prominent one and the most common mechanism responsible for the regulation of lamin assembly and disassembly [20]. Lamin phosphorylation at different sites is related to specific roles during cell cycle, for instance, lamin A/C is highly phosphorylated at serine 22 (S22) and serine 392 (S392) during mitosis [21], leading to the nuclear disassembly. However, lamin A can also be found phosphorylated at serine 22 in interphase cells [22]. This high dynamic of lamin A/C during cell division might be one explanation for the presence of lamin A/C in the nuclear interior: after mitosis, the part of lamin A/C that does not get de-phosphorylated and subsequently re-incorporated in the nuclear periphery, remains phosphorylated in the nuclear interior [23]. During interphase, twenty in vivo lamin A/C phosphorylation sites had been identified, comprising eight lamin A phosphate high turn-over sites. From those eight sites, single-point mutations mimicking phosphorylation at S22, S392, and the double mutation at serines 404/407 (S404/407) showed increased lamin A signal in the nucleoplasm, compared to wild type lamin A [24]. Furthermore, lamin A phosphorylated at S22 and S392 in the nucleoplasm was shown to bind to genomic regions with characteristics of gene enhancers, revealing a new important role for the intranuclear lamins [25].

Nucleoplasmic phosphorylated lamins may have unexplored functions, distinct from the ones in the nuclear lamina, which can be regulated through the possibility of new lamin complex assemblies. The best described lamin A/C nucleoplasmic interaction is with the lamin associated peptide 2 alpha (LAP2α) protein. While lamin A/C at the nuclear periphery is connected with heterochromatin through lamina-associated domains (LADs) being therefore associated with gene repression [26], the nucleoplasmic lamin A/C was found to also be associated with euchromatic genomic regions outside of LADs, overlapping with the regions that are also bound by Lap2α. Furthermore, cells derived from LAP2α knockout mice exhibit significantly reduced levels of lamin A/C in the nucleoplasm [18,27]. Emerin is a binding partner of prelamin A isoforms, and prelamin A accumulation in human fibroblasts was shown to alter Emerin localization [28]. Moreover, an important lamin A/C protein complex is formed by nuclear myosin 1 (NM1) and emerin [29,30], where lamin A/C knockdown decreases emerin-NM1 binding, indicating the role of lamin A/C in stabilizing their interaction. Another study suggests that lamin A/C and NM1 interaction modulates heat shock protein 70 (HSP70) transcriptional responses during heat shock [31]. However, the molecular mechanisms of interaction between lamin A/C and NM1 are still unknown. NM1 is the nuclear isoform of the cytoplasmic Myosin-1C (myo1c); it localizes to transcription sites in an RNA-dependent manner [32,33,34,35,36] and is likely to function as an actin-based motor that activates transcription [37]. In the plasma membrane, myo1c interacts with high affinity to the headgroup of phosphatidylinositol-4,5-bisphosphate (PI(4,5)P2, or PIP2) [38]. Furthermore, both myo1c and PI(4,5)P2 are localized in actin-rich structures, where PI(4,5)P2 is known to directly regulate several cytoskeletal proteins such as actin-related protein 2/3 (Arp2/3) complex activators or profilin [39]. In the nucleus, PI(4,5)P2 has also been described to bind profilin I [40], and the participation of PI(4,5)P2 and actin in the chromatin remodeling SWItch/Sucrose Non-Fermentable (SWI/SNF) complex recruitment was also reported [41]. Phosphatidylinositols (PIs) are lipid second messengers crucial for cell signaling. PI(4,5)P2 is the most well-studied nuclear phosphoinositide (PIP)—molecules derived from the PI metabolism, which involves the addition or removal of phosphate groups to the inositol ring (myo-conformer headgroup) [42]. PI’s inositol head group can be phosphorylated at 3′, 4′ and 5′ positions, forming then seven different phosphoinositides (PIPs). In the nucleus, 168 proteins harboring phosphoinositide binding domains have been identified by a proteomic approach based on neomycin extraction [43]. These proteins are involved in cell division, cell signaling, and transcription, showing the importance of phosphoinositides in many nuclear processes. We have previously shown that both NM1 [44] and PI(4,5)P2 [45] are required for RNA polymerase I (Pol I) transcription. PI(4,5)P2 affects RNA Pol I transcription by changing the conformation of upstream binding factor (UBF) and thus affecting its binding to ribosomal RNA (rRNA) genes [38]. It has been reported that NM1 interacts in an unclear way with RNA polymerase II (Pol II) [46,47]. This NM1-Pol II interaction is dependent on PI(4,5)P2 binding and anchoring NM1 in the nucleus. Moreover, mutated NM1 (K908A) that is unable to bind PI(4,5)P2, loses its association with Pol II, which leads to significantly reduced Pol II transcription output [48]. In a high-resolution quantitative mass spectrometry study using SILAC-based proteomic approach, 405 PIP-binding proteins were identified, of which 141 were PI(4,5)P2 interactors. Lamin A/C was identified in the initial total list of potential PIP interactors in all experiments including PI and all seven PIPs as baits. This set of proteins was highly consistent between experiments. [49]. Although Lamin A/C was not included in the final filtered subset of PIP interactors, these findings gave us a hint to its potential involvement in PIP-containing complexes.

Hence, we questioned whether lamin A/C, NM1, and PI(4,5)P2 are associated together in a complex. In this study, using biochemistry and super-resolution microscopy techniques, we report that lamin A/C and PI(4,5)P2 form a complex in the cell nucleus together with NM1, and that the assembly of this complex is modulated by lamin A/C phosphorylation status. Lamins were shown to directly bind to nuclear proteins, DNA [50], chromatin, nucleosomes, and histones [51,52]. Here, we show for the first time that lamin A/C also directly binds to phospholipids. As the functions and interaction partners of intranuclear lamins remain largely unknown, this finding suggests new possible ways of nucleoplasmic lamin A/C regulation, function, and importance for the formation of functional nuclear microdomains.

## 2. Materials and Methods

### 2.1. Cell Culture and Transfections

Human cervical carcinoma (HeLa) and osteosarcoma (U2OS) cells were grown in Dulbecco’s modified Eagle’s medium (Sigma-Aldrich, Prague, Czech Republic) supplemented with 10% fetal bovine serum (FBS) and 1% antibiotics (penicillin, streptomycin), at 37 °C in 5% CO_2_ humidified atmosphere. Suspension HeLa cells were grown in S-MEM supplemented with 5% FBS and 1% antibiotics at 37 °C in 5% CO_2_ humidified atmosphere. Cells were transfected with Lipofectamine 2000 (Invitrogen), according to the respective manufacturer’s instructions. B-lymphocytes from a Hutchinson–Gilford progeria syndrome (HGPS) patient (AG10587) were obtained from the Coriell Institute. Cells were grown in Gibco Roswell Park Memorial Institute (RPMI) medium (Sigma-Aldrich, Prague, Czech Republic) with 2 mM L-glutamine, supplemented with 15% fetal bovine serum (FBS), at 37 °C in 5% CO_2_ humidified atmosphere. Patient clinical information: HGPS [patient AG10587] with mutation in G608G—progerin—male, B-lymphocytes—Afro-American; clinical signs of progeria: alopecia, arthritis, skin changes, coxa valga, and angina. The donor has 6 times the normal level of hyaluronic acid in his urine. For direct stochastic optical reconstruction microscopy (dSTORM) analysis, U2OS cells were plated one day before staining in ~50% confluence on the high-precision 12 mm round coverslips treated with Hellmanex, sonicated, washed, dried, and sterilized.

### 2.2. Constructs and Expression of Recombinant Proteins

Expression vectors for GFP-tagged full length pre-lamin A, either wild-type or mutated at high-turnover phosphorylation sites were used. For each site, two types of mutations were introduced: phosphomimetic (from serine—S to aspartic acid—D) or phosphorylation-deficient (from serine—S to alanine—A). Lamin A wild-type and point mutations of individual phosphorylation sites (S22D; S22A; S22A, S392D; S22D, S392A; S628D; S628A; S404/407D; S404/407A; and S22D, S392D) were cloned in pEGFP-C1 construct with green fluorescence protein (GFP) and a c-myc tag as previously described [24]. WT-NM1 and K908A mut-NM1 were cloned with a Flag and Strep tag as described [48]. GFP-lamin A-WT and GFP-lamin A-phosphorylation mutant constructs were transfected in HeLa cells using Lipofectamine 2000 (Invitrogen) following the manufacturer’s protocol. Upon 24 h of transfection, cells were trypsinized, pelleted, and washed in ice cold phosphate buffer saline (PBS), and proceeded to cell lysis (see below in pull down and co-immunoprecipitation methods). 

### 2.3. Antibodies and Proteins

The following primary antibodies were used: anti-PI(4,5)P2 mouse monoclonal IgM antibody (Echelon Biosciences Inc., Salt Lake City, USA, clone 2C11, Z-A045); anti-lamin A/C mouse monoclonal IgG2a isotype (Sigma-Aldrich, Prague, Czech Republic, clone 4C11, SAB4200236), used for immunoblotting and immunofluorescence; anti-lamin A/C mouse monoclonal IgG (CJ Hutchinson, JOL2), used for immunoprecipitation assays; anti-NM1 rabbit polyclonal IgG (Sigma Aldrich, Prague, Czech Republic, M3567); anti-c-myc mouse monoclonal IgG1 kappa (Thermo Fisher Scientific, Waltham, MA, USA, clone 9E10, 13-2500); anti-GFP rabbit polyclonal IgG (Abcam, ab6556); control mouse anti-IgM (Abcam, Cambridge, UK, ab18400); control mouse anti-IgG (Abcam, Cambridge, UK, ab81032); control rabbit anti-IgG (Abcam, Cambridge, UK, ab46540).

Secondary antibodies used: goat anti-mouse IgM (u-chain specific) antibody conjugated with Alexa Fluor 555 (Invitrogen, Carsbad, USA, A21426), IRDye 680 donkey anti-mouse IgG (H+L) antibody (LI-COR Biosciences, Lincoln, USA, 926-68072), IRDye 680 donkey anti-rabbit IgG (H+L) antibody (LI-COR Biosciences, Lincoln, USA, 926-68073), IRDye 800 donkey anti-mouse IgG (H+L) antibody (LI-COR Biosciences, Lincoln, USA, 926-32212), IRDye 800 donkey anti-rabbit IgG (H+L) antibody (LICOR Biosciences, Lincoln, USA, 925-32213), IRDye 800 Goat anti-Mouse IgM (μ chain specific) antibody (LI-COR Biosciences, Lincoln, USA, 926-32280).

Recombinant human lamin A protein, with >90% purity, was purchased from Abcam, Cambridge, UK (ab83472). Dodecyl-sulfate polyacrylamide gel electrophoresis (SDS-PAGE) was used to verify protein purity.

### 2.4. Pull down and Co-Immunoprecipitation

Cells were trypsinized, pelleted, and washed with ice cold phosphate-buffered saline (PBS) 3 times. After the last wash, PBS was removed and cell lysis was made by adding 1× RIPA buffer (50 mM Tris-HCl pH 7.5, 150 mM NaCl, 1% NP-40, 0.5% deoxycholate, and complete protease inhibitors from F. Hoffmann-La Roche Ltd., Basel, Switzerland to the cell pellet, 30 min on ice. Then, the lysate was centrifuged for 15 min at 3500 rpm: the pellet containing cell debris was discarded. Protein concentration was calculated using bicinchoninic acid (BCA) protein assay. For the pull down (PD), agarose beads coated with phospholipids (Echelon Biosciences Inc., Salt Lake City, USA) were used: 60 µL of beads were washed 3 times with 1× RIPA buffer, followed by blocking the beads with 3% bovine serum albumin (BSA) in PBS for approximately 30 min at room temperature. The blocking solution was removed, and the protein lysate was added (1 mg/mL) and incubated for at least 2 h at 4 °C in a rotator. Then, the beads were again washed 3 times with PBS. The protein elution from the agarose beads coated with phospholipids was performed by boiling with 1x Laemmli buffer. For co-immunoprecipitation (Co-IP): 60 µL of magnetic protein G-beads (to IP lamin A/C and NM1) or magnetic protein L-beads (to IP PI(4,5)P2 ) was used. Beads were washed 3 times with 1× RIPA buffer, then 5 µg of antibody in 100 µL of PBS was added to the beads. The beads with the antibody were incubated for 2 h at 4 °C in a rotator, followed by 3 washes with PBS containing 0.05% Tween 20 (PBS-T) and 1 time with PBS. The protein lysate (1 mg/mL) was added to the beads and incubated for 2 h at 4 °C in the rotator. Beads were washed 3 times with PBS-T and 1 time with PBS; then, proteins were eluted by boiling with 1× Laemmli buffer. Input represents 20% of the total protein used for the PD/Co-IP.

### 2.5. Western Blot

Approximately 40 µg of protein lysate was loaded into a 10% polyacrylamide gel (unless stated otherwise) and separated by SDS-PAGE. Western blots and transfers were carried out following standard protocols in a semidry transfer system (Bio-Rad) to nitrocellulose membranes. Next, membranes were blocked for 1 h with 5% BSA in PBS with 0.05% Tween (*v*/*v*), followed by probing with relevant antibodies for 1 h at room temperature. Secondary antibodies conjugated with infrared dye (IRDye) were used (LI-COR Biosciences, Lincoln, NE, USA). Blots were developed using the Odyssey Infrared Imaging System (LI-COR Biosciences, Lincoln, NE, USA).

### 2.6. Protein-lipid Overlay Assay

Membranes spotted with phosphoinositides—PIP strips (Echelon Biosciences Inc., Salt Lake City, UT, USA, #P-6001)—were incubated, with 2.5 µg of pure lamin A in 5 mL of PBS for one hour in a shaker at room temperature. Strips were washed 3 times with PBS, blocked with 3% BSA in PBS followed by 3 washes with PBS. Strips were then immunoblotted with lamin A/C antibody for 1 h at room temperature. Membranes were incubated with goat/donkey anti-mouse IgG secondary antibodies conjugated to infrared dye (IRDye). The signal was detected using an Odyssey Infrared Imaging System (LI-COR Biosciences, Lincoln, NE, USA).

### 2.7. Cellular Fractionation

This protocol was adapted from the previously described Lamond lab protocol for cellular fractionation [53].

Suspension HeLa cells were grown until confluence in 500 mL of S-MEM supplemented with 5% FBS, in 5% CO_2_ humidified atmosphere. Cells were centrifuged for 7 min at 1500 rpm and the pellet was washed 3 times with PBS (1000 rpm for 5 min). The cell pellet was re-suspended in 5 mL of a cold buffer containing 10 mM HEPES pH 7.9, 1.5 mM MgCl_2_, 10 mM KCl, 0.5 mM DTT, protease inhibitors (cOmplete EDTA-free, Roche, Prague, Czech Republic). The cells were kept in the buffer for 5 min on ice. The cellular membrane was then broken using a Dounce homogeneizer. The dounced cells were centrifuged at 1000 rpm for 7 min at 4 °C, retaining the supernatant as cytoplasmic fraction and the pellet as nuclear fraction. From this point, we could either keep the nuclear pellet to subsequently prepare nuclear extracts, or we could continue for nuclear fractionation. For nuclear extracts, the nuclear pellet was re-suspended in 3 mL of a solution containing 0.25 M sucrose, 10 mM MgCl_2_ and cOmplete EDTA-free. The pellet re-suspended in that solution was then placed on the top of another solution containing 0.88 M sucrose, 0.5 mM MgCl_2_ and cOmplete EDTA-free, and centrifuged for 15 min at 4 °C at 3500 rpm; the pellet was kept as the nuclear fraction. This nuclear pellet was then dissolved in 1× RIPA buffer and sonicated. For sub-nuclear fractions, the pellet from the centrifuged dounced cells was re-suspended in 3 mL of a solution containing 0.25 M sucrose, 10 mM MgCl_2_ and cOmplete EDTA-free, and carefully placed over another sucrose buffer containing 0.35M sucrose, 0.5 mM MgCl_2_ and cOmplete EDTA-free. After this step, the nuclear pellet was resuspended in 3 mL of sucrose buffer: 0.35 M sucrose, 0.5 mM MgCl_2_ and cOmplete EDTA-free, and sonicate for 10 cycles of 10 s with 10 s off in between each cycle, in an ice bath. Trypan blue was added to a few microliters of the sonicated nuclei and checked under the microscope in order to determine the extent of the lysis. The sonicated sample was gently placed over 3 mL of the sucrose buffer 0.88 M sucrose, 0.5 mM MgCl_2_ and cOmplete EDTA-free, and centrifuged at 3500 rpm for 15 min, at 4 °C. The pellet is the nucleolei and the supernatant is the nucleoplasm. 1× RIPA buffer was added to each of the nuclear fractions, followed by centrifugation for 15 min at 4 °C; supernatant was kept and aliquoted.

The protein concentration from the nuclear pellet and nuclear fractions were measured using a BCA protein assay (Thermo Fisher Scientific, Waltham, MA, USA). 

### 2.8. Phosphatase Treatment

Nuclear extracts from HeLa cells (fractionated as described above), prepared without adding PhoStop phosphatase inhibitors, were treated with Lambda protein phosphatase (λPP) (NE BioLabs, Ipswich, MA, USA) according to manufacturer’s recommended protocol as follows: to the sample in a total volume of 40 µL, 5 µL of 10× NEBuffer +5 µL of 10 mM MnCl_2_ was added. Then, 2 µL of Lambda PP was added. The reaction was incubated for 40 min at 30 °C (in the shaker at 300 rpm). Control and dephosphorylated nuclear extracts were then separated by two-dimensional electrophoresis (described below).

### 2.9. Indirect Immunofluorescence, Confocal Microscopy and dSTORM

GFP-tagged lamin A-WT was transfected into U2OS cells, in 13 mm glass coverslips. Twenty-four hours after transfection, cells were washed 2 times in PBS for 4 min, at room temperature (RT). Subsequently, cells were fixed and permeabilized using 3% formaldehyde (PFA) and 0.1% Triton X-100 in PBS, for 20 min at RT. Then cells were washed 3 more times with PBS for 10 min and blocked with 5% BSA in PBS for 30 min at RT. After this step, the coverslips were transferred to a wet chamber, where 50 µL of the diluted primary antibody were added on the top of each coverslip, for at least 1 h at RT. After 1 h, the primary antibody was removed and cells were washed for 3 times at RT with PBS-0.05% Tween (PBS-T), followed by the addition of the secondary fluorescent antibodies for 1 h. Cells were then washed 3 times with PBS-T. Coverslips were mounted in ProLong Gold anti-fade reagent with DAPI (Life Technologies, Carlsbad, CA, USA). Images were acquired in confocal microscope Leica TCS SP8 with 405 nm and 561 nm laser excitation wavelengths using 63× (NA 1.4) immersion oil objective in Leica advanced fluorescence software (LAS AF).

dSTORM was performed as previously described [54,55]. The cells were washed twice with PBS (pH 7.4) and fixed for 30 min in 2% PFA in PBS, washed 3 times for 5 min with PBS, then permeabilized in 0.1% Triton X-100 in PBS for 20 min, washed 3 times for 5 min with PBS and blocked in filtered 5% BSA in PBS for 30 min. Cells were incubated for 45 min with primary antibodies diluted in 5% BSA in PBS, washed 3 times for 5 min in PBS and incubated for 30 min with secondary antibodies diluted in 5% BSA in PBS. Then the cells were washed 3 times for 5 min in PBS, post-fixed for 15 min in 2% PFA in PBS, and washed 3 times for 5 min in PBS. All procedures were performed at RT and the cells were stored in PBS. Coverslips with cells were mounted in the Chamlide chamber (Live Cell Instrument, Namyangju, Republic of Korea) and covered with imaging buffer (PBS pH 7.4, 50 mM MEA). Single-molecule localization (SML) data were acquired by Zeiss Elyra PS.1 equipped with HR Diode 642-150 and HR DPSS 561-200 lasers, Alpha Plan-Apochromat 100×/1.46 oil DIC M27 Elyra objective and Andor EM CCD iXon DU 897 camera, and ZEN Black 2.1 SP3 software (Zeiss, Oberkochen, Germany). AF647 and AF555 photo-switching was achieved by HiLo illumination and TIRF HP FOV with 100% power of 642 nm or 561 nm laser, and the signal was acquired via MBS 642 + EF LP 655 and MBS 561 + EF BP 570–620/LP 750 filters, respectively. Exposure time was 40 ms and EM gain was 300 for both channels.

SMLs were calculated in 2D by ZEN Black 2.1 SP3 software (Zeiss, Oberkochen, Germany) using x,y 2D Gauss fit with point spread function (PSF) half width 177.9 nm, peak mask size 9 pixels, and peak intensity to noise 6 and accounted for overlap in 2D with max cluster size 10. SMLs were rendered in ZEN software with 10 nm/px resolution and 1× PSF expansion factor. The data were model-based drift corrected in ZEN. Two channels were aligned with the affine fit and localization coordinates were exported as text files. Text files were converted into csv files and imported using self-written macro into the ImageJ2 plug-in ThunderSTORM and visualized by normalized Gaussian method. Random rotated localizations and images were created by swapping x and y coordinates. Localization precision was measured by ZEN software.

Randomly distributed SMLs were generated using ThunderSTORM ImageJ2 plug-in with respect to the number of frames, areas and molecular densities of the real data. Other parameters of the simulator were kept default. Nearest neighbor distances (NNDs) were calculated in ThunderSTORM using self-written macro, radius step 50 nm and step count 10. Results were imported into Excel, normalized by the number of localizations in each acquisition and displayed as the normalized distributions of NNDs with error bars representing the standard error of the mean (SEM). Mode (peak) NNDs were calculated from the distributions in Excel (Microsoft Office 2019, 64-bit). The data were statistically evaluated, and the Tukey whisker graphs were created in Prism (GraphPad, Prism 5, version 5.04). Statistical comparison was performed in Prism (GraphPad, Prism 5, version 5.04) using paired, one-tailed (NND analyses) or two-tailed (CT analyses) *t*-test for the normally distributed data tested by Kolmogorov–Smirnov test. Statistical significance was expressed as follows: * *p* < 0.05; ** *p* < 0.01; *** *p* < 0.005.

### 2.10. Two-Dimensional Electrophoresis (2D-E)

Two-dimensional gel electrophoresis was performed as previously described [56] with a few modifications. HeLa cells grown on 10 cm plastic cell culture dishes and transfected with WT-NM1, K908A mut-NM1, GFP-tagged lamin A-WT, and lamin A-phospho-mutants were trypsinized, washed with PBS, lysed by adding RIPA buffer, and sonicated. Proteins were immunoprecipitated using GFP antibody (for the case of WT-lamin A and lamin A phospho-mutants) or pulled down through the Strep Tag for the case of mut-NM1 and wt-NM1. Protein elution was performed in a solution containing urea (7 M), thiourea (2 M), CHAPS (4% *w*/*v*), Tris pH 9.5 (20 mM), pharmalite (2% *v*/*v*), DTT (40 mM), protease inhibitors. A rehydration buffer containing urea (8 M), CHAPS (2% *w*/*v*), bromophenol blue (0.002%), DTT (10 mM), pharmalite (2% *v*/*v*) was added to the protein solutions. The solution of proteins in the rehydration buffer (approximately 250 µg of protein) was then applied to 7 cm pH 3.0 to 10.0 immobilized pH gradient (IPG) strips for overnight (about 16 h) rehydration. Isoelectric focusing of the IPG strips was performed on a Multiphor II. Before running the second dimension, IPG strips were equilibrated in two different equilibration solutions for 15 min each. First equilibration solution contained Tris-HCl pH 8.8 (250 mM), urea (6 M), glycerol (30% *v*/*v*), SDS (0.02% *v*/*v*), DTT (65 mM), and bromophenol blue (0.002%). Second equilibration solution contained Tris-HCl pH 8.8 (250 mM), urea (6 M), glycerol (30% *v*/*v*), SDS (0.02%), iodoacetamide (135 mM), and bromophenol blue (0.002%). The second dimension was performed in a 10% SDS polyacrylamide gel with the equilibrated strip on the top of it and sealed with an agarose gel containing bromophenol blue, followed by electrophoresis. Proteins were transblotted (Trans-Blot Semi-Dry Transfer Cell, Bio-Rad Laboratories, Prague, Czech Republic) to a nitrocellulose membrane, and blocked in 3% BSA in PBS for 1h. Membranes were then immunoblotted with lamin A/C and GFP antibodies.

### 2.11. Data Analysis

For each experiment, a minimum of three independent experiments were carried out and the mean and SEM calculated. The quantification of the immunoprecipitation experiments was performed by normalizing the signal from immunoprecipitated proteins to the input (IP/input). The graphs next to the Western blot figures show the average of the total signal detected (in arbitrary units). For confocal microscopy images, we designed a macro for the nuclear segmentation and for quantification of the PI(4,5)P2 pattern, measuring total area, total intensity, number of foci per nucleus, and average area of each PI(4,5)P2 foci per nucleus. The Manders’ overlap coefficient was calculated using ImageJ software (version 2.1.0./1.53f51). For the analysis of variables following normal distribution, Student *t*-test (GraphPad Software, Prism 5, version 5.04) was performed. Differences were considered significant when *p* ≤ 0.05. The 2D-E plot profiles were obtained in ImageJ software (version 2.1.0./1.53f51).

## 3. Results

### 3.1. Lamin A/C, but Not Progerin, Is in Complex with Nuclear PI(4,5)P2

**Figure 1:**
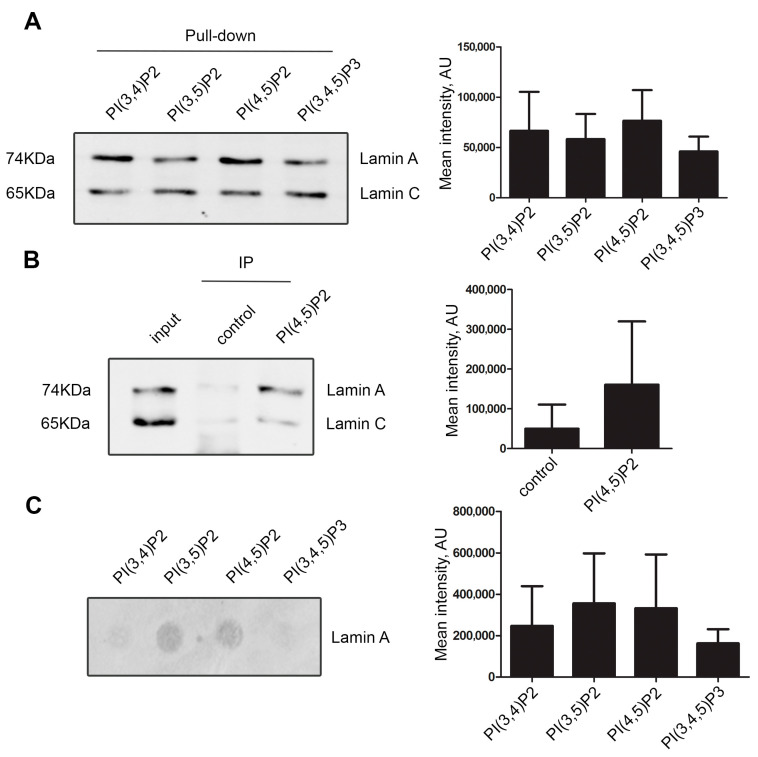
Lamin A/C interacts with nuclear phosphoinositides. (**A**) Pull-down of nuclear extract proteins using agarose beads coated with various phosphoinositides showing the interaction of lamin A/C to PI(3,4)P2, PI(3,5)P2, PI(4,5)P2, and to PI(3,4,5)P3. The graph represents average mean intensity of lamin A band in each phosphoinositide pull-down. There was no statistically significant difference in the lamin A binding between particular phosphoinositides. (**B**) Immunoprecipitation of lamin A/C by anti-PI(4,5)P2 antibody from HeLa cell nuclear extracts. On the left—representative immunoblot stained with anti-lamin A/C antibody; on the right—quantification. Input represents 20% of the total amount of protein used for the immunoprecipitation. The graph represents average mean intensity of lamin A band in control and IP. (**C**) Overlay assay showing direct binding of lamin A/C to PI(3,4)P2, PI(3,5)P2, PI(4,5)P2, and with less affinity to PI(3,4,5)P3. The PIP-strip—a hydrophobic lipid-containing membrane—was incubated with 2.5 µg of pure lamin A in 5ml of PBS, followed by immunoblotting with anti-lamin A/C antibody. On the left—a representative blot image; on the right—summarized data on the respective dot intensity. All experiments shown in A, B, and C were reproduced at least 3 times.

To test whether lamin A/C interacts with nuclear phosphoinositides, we pulled-down nuclear proteins from HeLa cells nuclear extracts using agarose beads coated with PI(3,4)P2, PI(3,5)P2, PI(4,5)P2, and PI(3,4,5)P3. Our results showed that lamin A/C interacts with all phosphatidylinositol-bisphosphates and triphosphate (Figure 1A and Supplementary Figure S1A). Due to our long-standing interest in PI(4,5)P2, we verified the interaction by co-immunoprecipitation of nuclear proteins using anti-PI(4,5)P2 antibody. Immunoblotting with anti-lamin A/C antibody (Figure 1B) clearly revealed PI(4,5)P2 as an interactor of lamin A/C. Interestingly, while the amount of lamin C is higher than lamin A in the input, the lamin A is relatively enriched in the PI(4,5)P2 pull-down. Some studies have already pointed to distinct properties and functions lamin A and lamin C related to the differences in their sequences and post-translational modifications patterns (reviewed in [57]); here, we observe another manifestation of these differences. To find out whether the interaction between lamin A and phosphoinositides was direct, we performed an in vitro protein–lipid overlay assay using purified recombinant lamin A and PIP strips, which are hydrophobic membranes spotted with various pure phosphoinositides (100 pmol per spot). The purity and correct size of the recombinant lamin A protein was verified by SDS-PAGE and Coomassie staining (Supplementary Figure S1B). The result demonstrated direct binding of lamin A to PI(4,5)P2 and PI(3,5)P2, and to a somewhat lesser extent, although not statistically significant, to PI(3,4)P2 and PI(3,4,5)P3 (Figure 1C and Supplementary Figure S1C).

**Figure 2:**
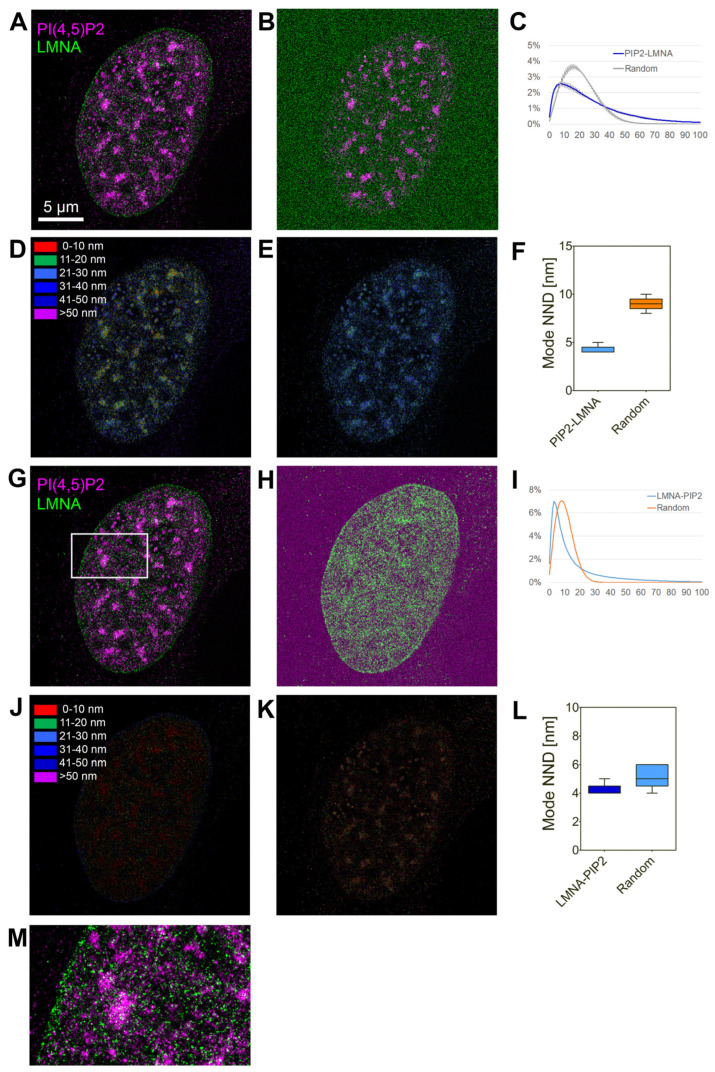
Lamin A/C and PI(4,5)P2 co-distribute non-randomly in the cell nucleus. (**A**) Lamin A/C indirectly immunolabelled with AF647 (green) and PI(4,5)P2 indirectly immunolabelled with AF555 (purple) in U2OS cells was imaged by dSTORM and visualized as normalized Gaussian fit of the PSF into the individual localizations, merged image. (**B**) Merged image of PI(4,5)P2-AF555 and lamin A/C-AF647 randomized by generating randomly distributed lamin A/C-AF647 signal. (**C**) Normalized distribution of the NND between PI(4,5)P2-AF555 and randomly generated lamin A/C-AF647 localizations. (**D**,**E**) Color-coded pixel map of the NND of PI(4,5)P2-AF555 to lamin A/C-AF647 in the real (**D**) and randomly generated images (**E**). (**F**) The most frequent (Mode) NND between PI(4,5)P2-AF555 and randomly generated lamin A/C-AF647 localizations. (**G**) Lamin A/C indirectly immunolabeled with AF647 and PI(4,5)P2 indirectly immunolabelled with AF555 in U2OS cells was imaged by dSTORM and visualized as normalized Gaussian fit of the PSF into the individual localizations, merged image (same as (**B**)). (**H**) Merged image of PI(4,5)P2-AF555 and lamin A/C-AF647 randomized by generating randomly distributed PI(4,5)P2-AF555 signal. (**I**) Normalized distribution of the NND between lamin A/C-AF647 and randomly generated PI(4,5)P2-AF555 localizations. (**J**,**K**) Color-coded pixel map of the NND of lamin A/C-AF647 to PI(4,5)P2-AF555 in the real (**J**) and randomly generated images (**K**). (**L**) The most frequent (Mode) NND between lamin A/C-AF647 and randomly generated PI(4,5)P2-AF555 localizations. (**M**) Zoom-in to the boxed region in (**G**).

We further analyzed mutual localization of endogenous lamin A/C and PI(4,5)P2 in the cell nucleus using super-resolution dual-color dSTORM microscopy of U2OS cells immunolabelled with anti-lamin A/C and anti-PI(4,5)P2 antibody (Figure 2). The analysis of co-distribution was performed by evaluation of nearest-neighbor distance (NND) distribution in real images and generated randomly distributed single-molecule localizations (SMLs). The results show non-random localization of PI(4,5)P2 in close proximity to lamin A/C with the mode NND at 8 nm (Figure 2C,F) and non-random localization of lamin A/C in close proximity to PI(4,5)P2 with the mode NND at 4 nm. For the in cellulo visualization of spatial organization of lamin A/C and PI(4,5)P2, we used our previously developed visualization tool that enables color-coding of the pixels according to the pair-wise NND of the SMLs in one channel with the respect to SMLs in the second channel. Figure 2D shows the in cellulo map in which the pixels are color-coded according to NND of the PI(4,5)P2-AF555 to lamin A/C in the real image. In contrast, Figure 2E illustrates the increased NND in the randomly generated image. Figure 2J,K show the respective in cellulo NND maps for lamin A/C to PI(4,5)P2. 

Taken together, these results demonstrate that lamin A/C and PI(4,5)P2 interact and are localized in close proximity to each other in the cell nucleus.

**Figure 3:**
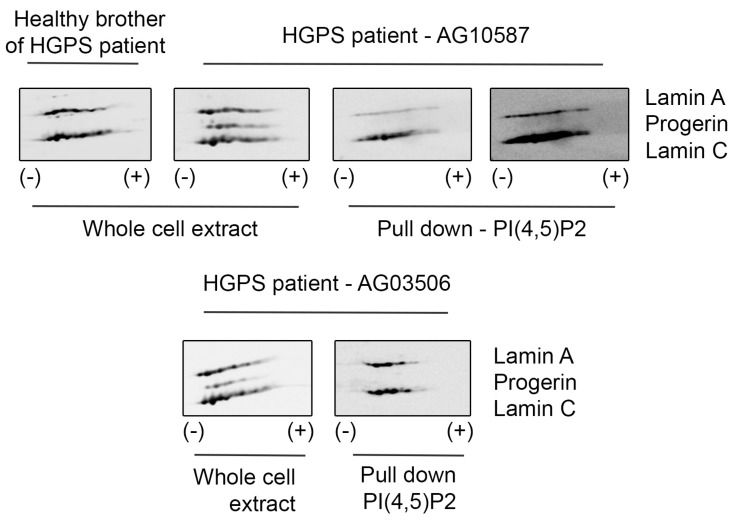
PI(4,5)P2 interacts with lamin A/C but not with progerin. B-lymphocyte cell extracts from HGPS patients and their healthy relative were separated by 2D electrophoresis. Progerin is visualized with lamin A/C antibody in the samples from whole cell extract. Whole cell extract represents 20% of the protein used for the pull down. When proteins from the same patient are pulled down with PI(4,5)P2, we can only see the interaction with lamin A/C, while progerin is not detected. Experiment was repeated using cells from two different HGPS patients.

Hutchinson–Gilford Progeria Syndrome (HGPS) is caused by de novo mutations in the LMNA gene, which activate an alternative pre-mRNA splice site, leading to the expression of progerin—a lamin A mutant lacking 50 amino acids in its globular tail domain. HGPS cells show increased apoptosis, impaired DNA damage repair, and cell cycle regulation defects, among other regulation deficiencies [58]. On the other hand, nuclear phosphoinositides, including PI(4,5)P2, have been described to be implicated in the signaling cascade that leads to the regulation of DNA repair in the nucleus [59,60,61]. Therefore, we would like to assess whether PI(4,5)P2 also interacts with progerin. For this purpose, we used B-lymphocyte cell lines derived from HGPS patients and their healthy relative. Interestingly, while PI(4,5)P2 interacts with lamin A/C, it does not interact with progerin (Figure 3). Fibroblasts from HGPS patients have been earlier reported to be slower in recruitment of DNA damage response proteins, indicating defective DNA repair pathways [62,63], and PI(4,5)P2 has been associated with DNA damage response. The lack of binding of PI(4,5)P2 to progerin might thus be part of this mechanism contributing to the respective normal vs HGPS phenotype. Moreover, we could also observe changes in PI(4,5)P2 localization upon DNA damage induced through ultraviolet (UV) radiation: irradiated cells, apart from showing more H2AX signal, also presented less PI(4,5)P2 foci per nucleus; however, those foci were significantly bigger (Supplementary Figure S2).

### 3.2. A Portion of Intranuclear Lamin A/C Interacts with NM1 in a PI(4,5)P2-Dependent Manner

**Figure 4:**
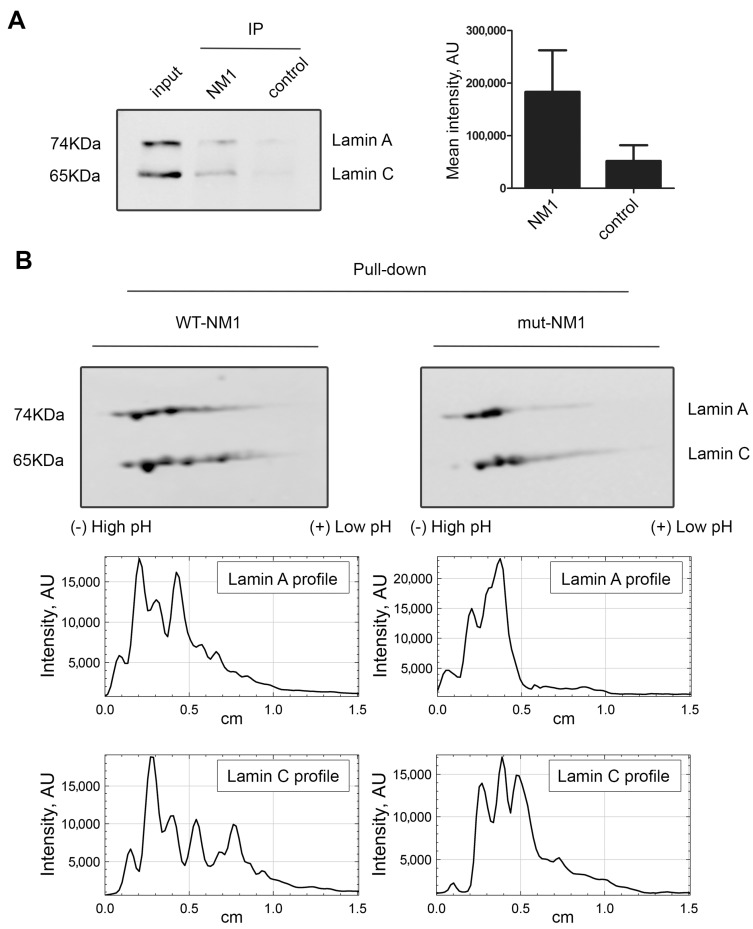
Lamin A binds to NM1 in a PI(4,5)P2-dependent manner. (**A**) Lamin A/C interacts with NM1. Immunoprecipitation (IP) of nuclear proteins by NM1 antibody shows its interaction with lamin A/C. On the left—a representative Western blot image; on the right—summarized data on the respective band intensity. *p* < 0.1. The experiment was reproduced 3 times. Input represents 20% of the total amount of protein used for the immunoprecipitation. (**B**) More acidic forms of lamin A are not bound by mut-NM1. Proteins were pulled down from nuclear extracts of HeLa cells expressing either WT-NM1 or mut-NM1 (incapable to bind PI(4,5)P2) using anti-Strep tag antibody. The proteins were separated by 2D-E followed by immunoblotting with lamin A/C antibody. Several more acidic lamin A forms were present only in complex with wild type NM1, but not with the mutant one. On the middle panel, there are the plot profiles of the 2D pattern of lamin A, and on the bottom, the plot profiles of lamin C. The experiment was performed twice.

Lamin A/C was identified together with nuclear myosin I (NM1) in emerin protein complexes isolated by affinity purification, ion exchange chromatography, and size exclusion chromatography [29]. In addition, our group have shown that NM1 also directly interacts with PI(4,5)P2 in the cell nucleus [48,64]. We hypothesized that lamin A/C, NM1, and PI(4,5)P2 can be part of the same complex. To assess the NM1 and lamin A/C interaction, immunoprecipitation using anti-NM1 antibody was performed and the co-immunoprecipitated proteins were separated by SDS-PAGE and transferred to nitrocellulose membrane. Immunoblotting with anti-lamin A/C showed that indeed lamin A/C is associated with NM1 in the cell nucleus (Figure 4A and Supplementary Figure S3).

To analyze the relation between lamin A/C, PI(4,5)P2, and NM1 in more depth, we used our previously designed constructs: wild type Flag-Strep-tagged NM1 (WT-NM1) or a mutant Flag-Strep-tagged NM1, which contains a mutation in the PH domain (K908A) that abolishes binding to PI(4,5)P2 (mut-NM1) [48]. HeLa cells were transiently transfected with WT-NM1 and mut-NM1, and pull-down via strep-tag was performed from the whole-cell extracts. The pulled-down proteins were separated by two-dimensional electrophoresis (2D-E) followed by immunoblotting with lamin A/C antibody. While lamin A/C pattern from the input of WT-NM1 and mut-NM1 cell lysates showed no difference (Supplementary Figure S4), the pattern of lamin A/C spots exhibited different distribution between pull-down of WT-NMI and mut-NM1 associated protein. Several lamin A/C spots towards the acidic side were missing in mut-NM1 complexes (Figure 4B), suggesting that the ability of NM1 to bind PI(4,5)P2 is essential for interaction with these lamin A forms.

**Figure 5:**
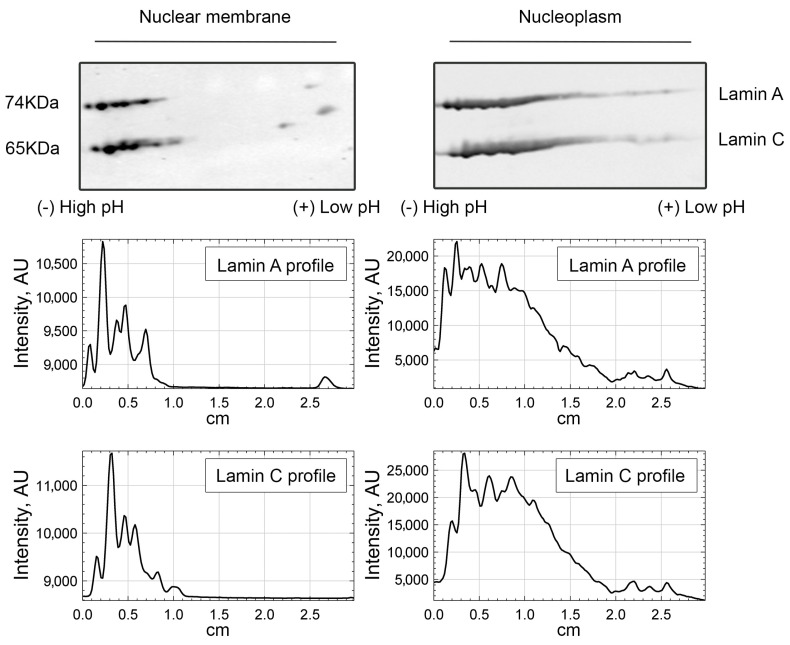
Phosphorylated lamin A/C isoforms are enriched in nucleoplasm. Two-dimensional electrophoresis of fractionated nuclear proteins from HeLa cells shows that the nucleoplasmic fraction contains more forms of lamin A/C with lower pI than the nuclear membrane fraction. Plot profiles underneath the blots represent the mean intensity of the signal along the protein 2D pattern, measured separately for lamin A and lamin C. The experiment was performed twice.

We then questioned what the lamin A/C pool that requires PI(4,5)P2 to interact to NM1 is. Since PI(4,5)P2 localizes to the internal part of the cell nucleus, and not to the lamina, we performed nuclear fractionation and compared the nucleoplasmic and nuclear membrane-enriched fractions separated by 2D-E. The results of immunoblotting by lamin A/C antibody showed that the more acidic forms of lamin A/C are enriched in the nucleoplasm, while barely detectable in the nuclear lamina fraction (Figure 5). Remarkably, these more acidic lamin A molecules present in the nucleoplasm are in the same range of pI (isoelectric point) as those lamin A/C forms that bind to NM1 in a PI(4,5)2-dependent manner. This suggests that even though A-type lamins both at the nuclear periphery and in the nuclear interior bind to NM1, a part of ones present in the nuclear interior require PI(4,5)P2 to do so. The subnuclear co-distribution of lamin A/C and PI(4,5)P2 was also assessed by confocal laser scanning microscopy. U2OS cells expressing GFP-tagged lamin A/C were immunolabeled with anti-PI(4,5)P2 antibody. Image analysis of whole nuclei in ImageJ using the Coloc2 plugin showed certain level of correlation between the lamin A/C–PI(4,5)P2 signal (Pearson’s correlation coefficient (PCC) is 0.4). To distinguish between the nuclear envelope (NE) and the nucleoplasmic signal, we developed a macro for nuclear segmentation. The results showed that the overlap of lamin A/C over PI(4,5)P2 signal is significantly higher (*p* < 0.0001) in the nucleoplasm (Manders’s coefficient (MOC) = 0.893) compared to the NE (MOC = 0.479) and also to the whole nucleus (MOC = 0.702) (Supplementary Figure S5).

These results show that A-type lamins associate with NM1 both in PI(4,5)P2-independent and PI(4,5)P2-dependent manner, the latter interaction occurring in the nuclear interior. 

### 3.3. Lamin A/C Phosphorylation Is Important for the PI(4,5)P2-Dependent Binding to NM1 

**Figure 6:**
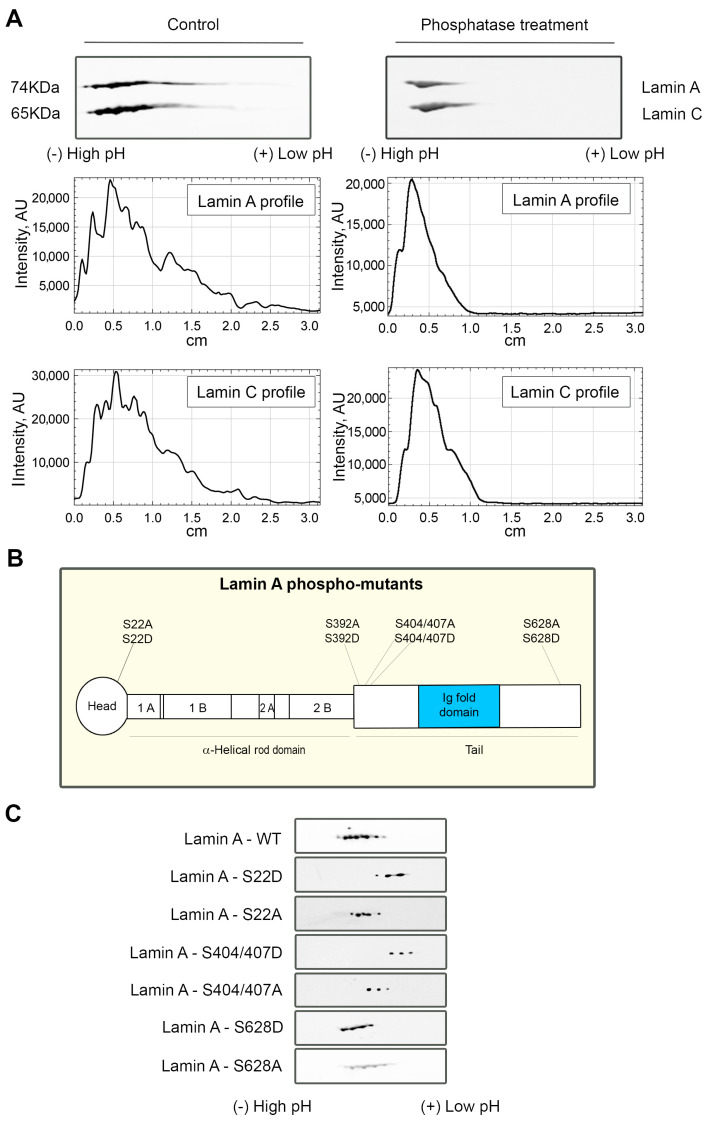
Phosphorylation status of lamin A/C plays a role in the complex assembly. (**A**) Nuclear extracts of HeLa cells were separated by 2D–E without any treatment (control, left panel) and after treatment with λPP (phosphatase treatment, right panel) and immunoblotted with lamin A/C antibody. The lamin forms with lower pI represent phosphorylated protein. Experiment was performed twice. (**B**) Scheme of lamin A molecule, displaying the position of mutated phosphorylated sites: S22 in the head domain, S392 and S404/407 in the rod domain, and S628 in the C-terminal tail of the protein. (**C**) Lamin A phospho-mutant 2D-E distribution showing that lamin A mutations affect the phosphorylation pattern of lamin A, causing a shift to the acidic side. HeLa cells were transfected with lamin A WT and lamin A phospho-mutants tagged with GFP. Immunoprecipitation with anti-GFP antibody was followed by separation in 2D–E and immunoblotting with anti-GFP antibody. The experiment was performed in two biological replicates.

Lamin A/C undergoes numerous post-translational modifications [65]; thus, we hypothesized that the more acidic forms of lamin A interacting with NMI in PI(4,5)P2-dependent manner could represent some of those modifications. It is known that some phosphorylations may lead to the partial depolymerization of the lamina and cause relocation of lamin A/C to the nucleoplasm [24]. As lamin A/C molecule contains over 40 phosphorylation sites [65], and phosphorylation is known to change the isoelectric point (pI) of proteins [66], we tested whether the more acidic lamin A/C forms observed that only bind to NM1 in the presence of PI(4,5)P2 correspond to the phosphorylated A-type lamins, which would also go in line with the nucleoplasmic localization of this complex. In order to test this hypothesis, proteins in HeLa nuclear extracts were dephosphorylated using lambda protein phosphatase (λPP), which has activity towards phosphorylated serine, threonine, and tyrosine residues. Extracts treated with λPP and control non-treated samples were subsequently separated by 2D-E and immunoblotted with lamin A/C antibody. The more acidic forms of lamin A/C disappear after the phosphatase treatment, indicating that the pI shift is caused by lamin A/C phosphorylation (Figure 6A).

Upon finding that lamin A/C phosphorylation status plays a role for the formation of the complex with NM1 and PI(4,5)P2, we attempted to specify the individual phosphorylation sites at lamin A molecule responsible for this interaction. We overexpressed GFP-tagged WT-lamin A and lamin A-phospho-mutants bearing phosphomimetic (from serine—S to aspartic acid—D) or phosphorylation-deficient (from serine—S to alanine—A) mutations (Figure 6B) in HeLa cells. The exogenous lamin A was immunoprecipitated from cell lysates with anti-GFP antibody, and isolated proteins were separated by 2D-E, followed by immunoblotting with anti-GFP antibody. We observed a major lamin A/C shift towards more acidic pH in some of the phospho-mutants mimicking the phosphorylation when compared to the mutation that inhibits the phosphorylation in the same residues (Figure 6C). These results point out that lamin A/C phosphorylation sites, or their clusters, might be involved to different extents in the formation of a PI(4,5)P2-dependent complex of lamin A/C and NM1.

## 4. Discussion

Lamins, the nuclear intermediate filament proteins, are important regulators of nuclear structural integrity as well as nuclear functional processes [2] such as DNA transcription, replication and repair, and epigenetic regulations. There are two structurally distinct lamin pools: in the nuclear lamina and in the nuclear interior, which have at least partially different specific functions. Cellular functions exerted by lamins are regulated by complex post-translational modifications and molecular interactions of lamins. In this study, we have found and biochemically characterized a novel and yet undescribed complex formed by phosphorylated lamin A/C, PI(4,5)P2, and NM1 in the nuclear interior.

It was previously reported in independent studies that both NM1 and PI(4,5)P2 might be lamin A/C interactors. Lamin A/C was identified together with nuclear myosin I (NM1) in emerin protein complexes [29], and it was found in the PIP-binding proteome as one of potential interactors in a total non-filtered list from all experiments [49]. Here, we show by targeted biochemical approaches than lamin A/C binds to PI(4,5)P2 directly, and also interacts with NM1. Two-dimensional gel electrophoresis (2D-E) allows us to separate the proteins not only by molecular mass but also by isoelectric point (pI), which changes in the presence of post-translational modifications (PTMs) in the protein. In this study, we pulled down proteins associated with wild type NM1 or mutated NM1 unable to bind PI(4,5)P2 and separated them by 2D-E, which allowed us to reveal a portion of lamin A/C molecules with lower pI that bound NM1 in a PI(4,5)P2-dependent manner. Treatment of nuclear extract proteins with lambda protein phosphatase (λPP) demonstrated that these more acidic lamin A/C forms represent a phosphorylated lamin A/C pool. Protein phosphorylation is a key regulatory process involved in most known cellular processes [67] affecting protein structural properties, stability, and dynamics, as well as regulating their binding to molecular partners [68]. While there are multiple phosphorylation sites on the lamin A/C molecule [69], the knowledge on the roles of lamin phosphorylation is rather limited. Effects of phosphorylation in specific lamin residues have been reported for the processes of nuclear envelope breakdown in mitosis [21,70] and apoptosis [71], lamina reorganization and reformation, regulation of nuclear size, mechanical properties and mechanosensing [72,73,74], and subnuclear distribution of lamins [24]. So far, only two studies have reported specific lamin interactions with binding partners modulated by lamin phosphorylation. Association between lamin B and ubiquitous transcription factor organic cation transporter 1(Oct1) was lost upon lamin B1 phosphorylation by c-Jun N-terminal kinases (JNK) upon stress conditions [75], and phosphorylation of S22 and S392 residues in lamin A/C enabled its association with genomic enhancer binding sites near active genes [25]. Thus, our results show for the first time a direct interaction of lamin A/C with a nuclear phosphoinositide, as well as lamin association with a protein binding partner—NMI—positively regulated by lamin phosphorylation.

We can hypothesize several possible mechanisms of the lamin A/C-NM1-PI(4,5) complex assembly. Binding of phosphoinositides to proteins has been reported to change the respective protein conformation and thus affect its interactions with the binding partners [76,77]. As NM1 binds to PI(4,5)P2 directly, this may cause NM1 conformational change enabling its binding to phosphorylated lamin. While charge-based binding of negatively charged PI(4,5)P2 to phosphorylated and thus also bearing negative charge lamin A/C is unlikely, we however cannot exclude PI(4,5)P2 binding to lamin A/C due to phosphorylation-related conformational change. Lamin phosphorylation may also affect its phase-separation properties [78,79], which could be another mechanism contributing to the formation of the complex. We previously hypothesized that PI(4,5)P2 might play a role in phase separation-driven nuclear processes [64], and it was recently shown that phosphoinositides are involved in formation of various biomolecular condensates [80]. Further studies will be needed to elucidate the structure of the complex and the exact mechanisms and dynamics of its assembly. Notably, our results show that binding between lamin A/C and NM1 occurs both in PI(4,5)P2-dependent and independent ways, with phosphorylated or less phosphorylated lamin, respectively. We also observe differences in interaction of lamin A and lamin C with PI(4,5)P2. We hypothesize that the formation of these lamin A/C-NM1 complexes in the cell nucleus regulated by PI(4,5)P2 presence and modulated by lamin phosphorylation represent a new mechanism of regulatory functions of lamin in the cell nucleus. 

Since PI(4,5)P2 [48], as well as NM1 [81,82], localize to the internal part of the cell nucleus, and not to the lamina, we hypothesized that the lamin A/C-NM1-PI(4,5)P2 complexes are formed by an intranuclear lamin pool. Our results of nuclear fractionation followed by 2D-E and of microscopy analysis confirmed this assumption. More detailed 2D-E analysis of phosphomimetic and phosphorylation-deficient lamin A mutants showed that phosphomimetic mutants mimic, to some extent, the pI distribution of the phosphorylated nucleoplasmic lamin, as well as the lamin forms that exhibit PI(4,5)P2-dependent binding to NM1. The large shift in certain phosphomimetic lamin A mutants might be explained by the fact that although we are mimicking phosphorylation or abolishing the phosphorylation at that specific residue of the mutation, we do not have control over the phosphorylation/dephosphorylation events that might happen in the other residues of the molecule upon transfection to the cells. In the case the phosphorylation in serines 404 and 407, the pI shift was more pronounced, possible due to adjacent high-turnover phosphorylated serines—S403 and S406—which might create a local phosphorylation boost manifested as a major pI shift towards the acidic side. Our results are mainly in line with the interphase localization of phosphorylated lamins reported by Kochin et al. [24], where single-point mutations mimicking phosphorylation at Ser22, Ser392, and the double mutation at Ser404/407 increased lamin A signal in the nucleoplasm, compared to wild type lamin A [24]. Our findings further contribute to the elucidation of the mechanisms of origin and maintenance of the intranuclear lamin pool. We have shown previously that binding to PI(4,5)P2 reduces the mobility of NM1 in the cell nucleus and thus PI(4,5)P2 might be a part of a scaffold for formation of functional nuclear microdomains. Binding of phosphorylated lamin to PI(4,5)P2 and NM1 might contribute to retention of phosphorylated lamin A/C in the nuclear interior, regardless of its origin either from interphase phosphorylation events or post-mitotic nuclear re-assembly.

Both PI(4,5)P2 and NM1 have been implicated in transcription regulation and chromatin remodeling [48,76,77,83,84]. Moreover, in our previous study, we demonstrated that the knockdown of NM1 reduces the transcription levels of Pol II and that the Pol II transcription can be rescued by the overexpression of wild type NM1 but not by the mutant NM1 that does not bind to PI(4,5)P2 [48]. While lamins in the nuclear lamina are associated with gene repression and bind transcriptionally inactive chromatin domains [26], the lamins in the nuclear interior have been shown to also associate with euchromatin [27] and bind active enhancers upon phosphorylation [25]. The existence of an NM1 and PI(4,5)P2 complex with phosphorylated lamin in the nuclear interior, described in this paper, opens new directions for the future studies of mechanisms of gene expression regulation exerted by intranuclear lamins.

Another possible functional role of the lamin A/C—NM1—PI(4,5)P2 complex might be involvement in DNA damage response processes. Both PI(4,5)P2 [59,60,61,85] and NM1 [86] have been associated with DNA damage response. Hutchinson–Gilford Progeria syndrome (HGPS), which is characterized mostly by its accelerated normal human aging conditions [87,88], is caused by de novo mutations in the *LMNA* gene that activate an alternative pre-mRNA splice site leading to the expression of progerin—a lamin A mutant lacking 50 amino acids in its globular tail domain. In HGPS cells, increased apoptosis, impaired DNA damage repair, and cell cycle regulation defects, among other regulation deficiencies, were observed [58]. Progerin accumulation leads to increased DNA damage and is manifested in the slower molecular response of the ataxia telangiectasia-mutated (ATM) and ATM- and Rad3-related (ATR) activation as well as phosphorylation of serine/threonine-protein kinase 1 and 2 (Chk1 and Chk2) [63,89]. ATM and ATR are members of the phosphatidylinositol 3-kinase-like family of serine/threonine protein kinases (PIKKs) and play important roles in the cellular response to DNA damage [90]. Interestingly, our pull-down data from cells derived from HGPS patients and their healthy relatives as control show that PI(4,5)P2 interacts with lamin A/C but not with progerin. Notably, progerin is lacking phosphorylation of S22 [91], which might be also connected to the lack of binding to PI(4,5)P2. Thus, our results suggest that the lamin A/C—PI(4,5)P2 complex described in this study may have a role in aging/DNA damage response processes.

Taken together, our study reveals a new interaction of lamin A/C with nuclear phosphoinositide PI(4,5)P2 and nuclear myosin 1 (NM1). In addition to direct interaction of lamin A/C with PI(4,5)P2 and overall binding between lamin A/C and NM1, phosphorylated lamin A/C forms complex with NM1 in a PI(4,5)P2-dependent manner in the interior of the cell nucleus. These findings advance our knowledge on the molecular interactions of intranuclear lamins and provide new basis for the future research of specific mechanisms by which lamins regulate gene expression, DNA damage repair, and organization of functional microdomains in the cell nucleus.

## Data Availability

Data are contained within the article or Supplementary Materials.

## Supplementary Materials

The following supporting information can be downloaded at: https://www.mdpi.com/article/10.3390/cells13050399/s1.
