# Supplementary material for: Lamin A/C and PI(4,5)P2—A Novel Complex in the Cell Nucleus
Source: Cells. 2024 Feb 25;13(5):399. doi: 10.3390/cells13050399 (PMC10931150; doi:10.3390/cells13050399)
Supplement: Supplementary file 1 [file cells-13-00399-s001.zip › cells-2676065-supplementary.pdf]

cells MDPI

Lamin A/C and PI(4,5)P2—A Novel Complex in the Cell Nucleus

Supplementary Figures

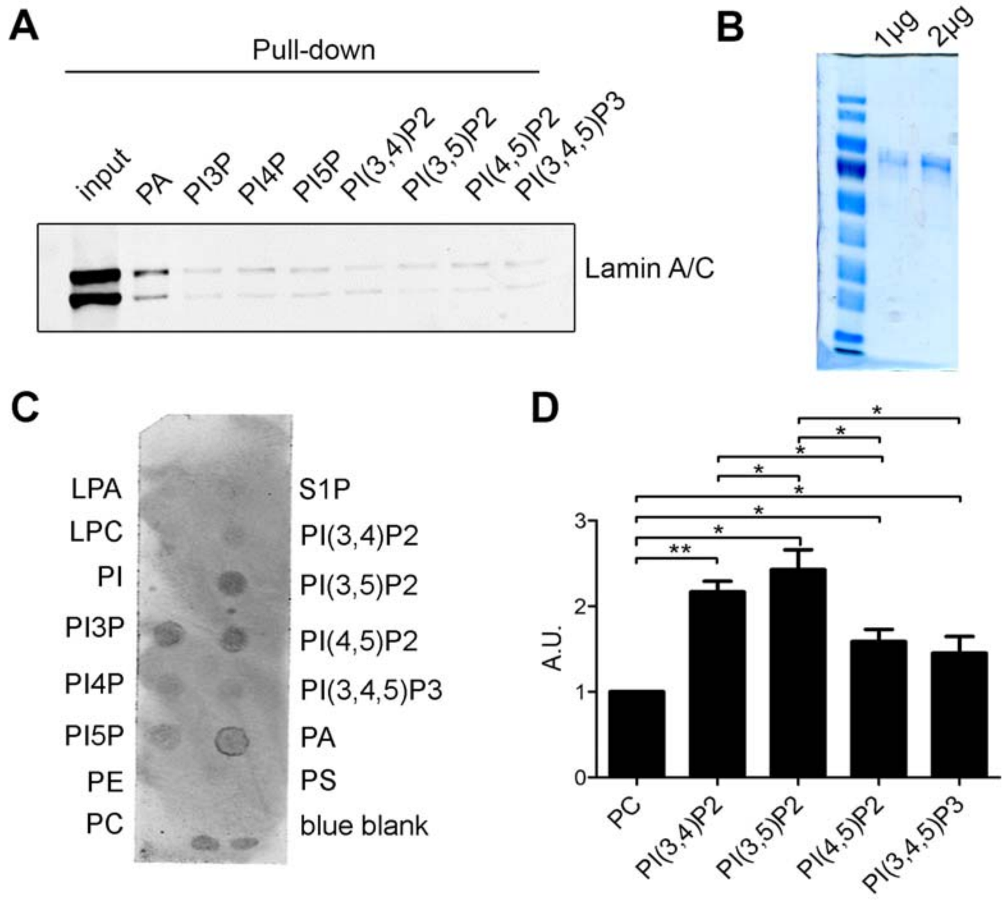

Supplementary Figure S1. (A) Pull-down of nuclear extract proteins using agarose beads coated with various phosphoinositides. Input is 20% of the total amount of protein used for the PD. Experiment was performed in 3 biological replicates. **(B)** Purified lamin A. Purity of lamin A was validated by SDS-PAGE followed by Coomassie staining. **(C)** PIP-strip incubated with purified lamin A followed by immunoblotting with anti-lamin A/C antibody. **(D)** Graph showing mean values of lamin signal on dots of PI bisphospates and triphosphate normalized to phosphatidylcholin (PC) dot signal, which was the lowest among all spots in all replicates.

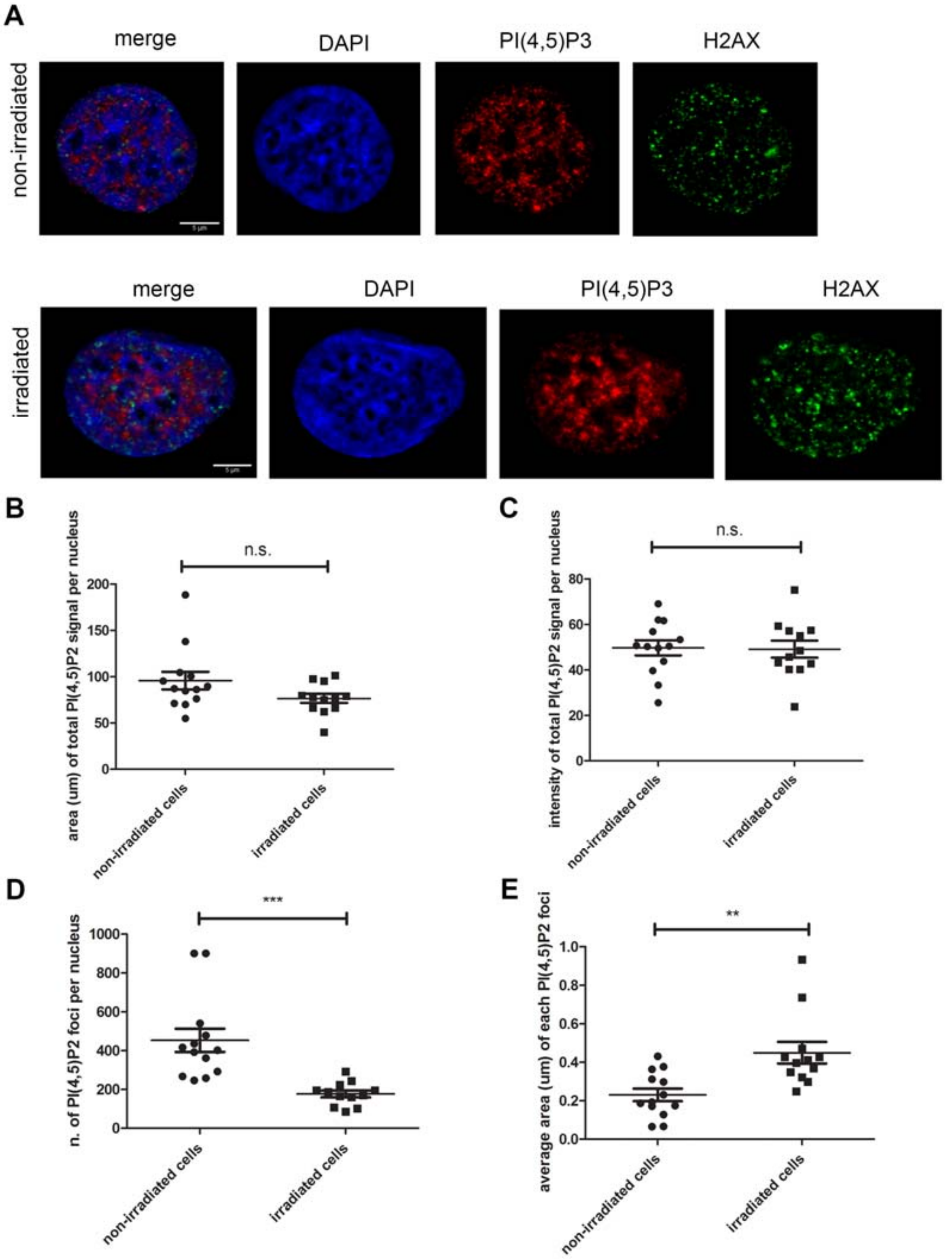

Supplementary Figure S2. DNA damage changes PI(4,5)P2 expression pattern. (A) Scanning confocal microscopy of non-irradiated and irradiated U2OS cells. Blue is Dapi, red is PI(4,5)P2, and green represents H2AX. Images represent a middle optical section of the nucleus. Bar, 5 µm. A macro for ImageJ was developed *in-house* in order to specifically analyze PI(4,5)P2 pattern. (B) Analysis of the total area of PI(4,5)P2 per nucleus, in control and irradiated cells. (C) Analysis of the intensity of the total PI(4,5)P2 signal per nucleus in control and irradiated cells. (D) Quantification of the number of PI(4,5)P2 per nucleus in control cells and in cells with induced DNA damage. € Average of each PI(4,5)P2 foci area per nucleus in control and DNA-damaged induced cells. n.s.: non-significant; $^{**}P < 0.01$; $^{***}P < 0.001$. Non-irradiated cells: n=13; irradiated cells: n=12.

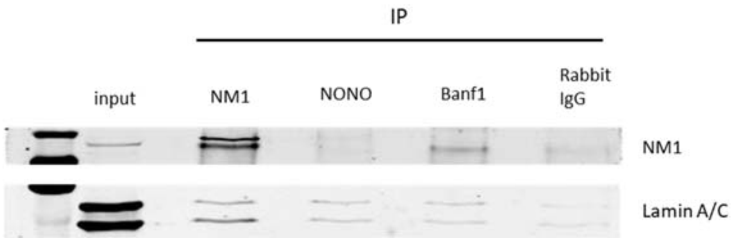

Supplementary Figure S3. Lamin A/C interacts with NM1. Immunoprecipitation (IP) of nuclear proteins by NM1 antibody shows its interaction with lamin A/C. Input is 20% of the protein amount used for the immunoprecipitation.

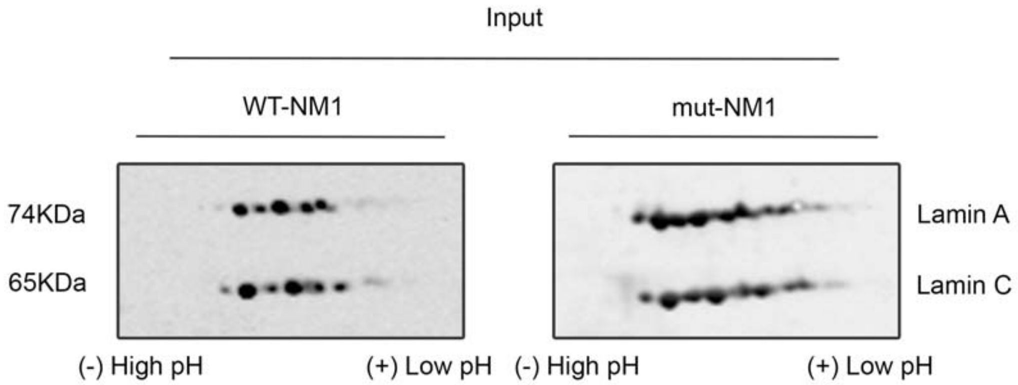

Supplementary Figure S4. Input from nuclear extracts of HeLa cells expressing either WT-NM1 or mut-NM1 (incapable to bind PI(4,5)P2) of Figure 4B pull down. Analysis of lamin A/C distribution pattern of WT-NM1 and mut-NM1 analyzed. The proteins were separated by 2D-E followed by immunoblotting with lamin A/C antibody.

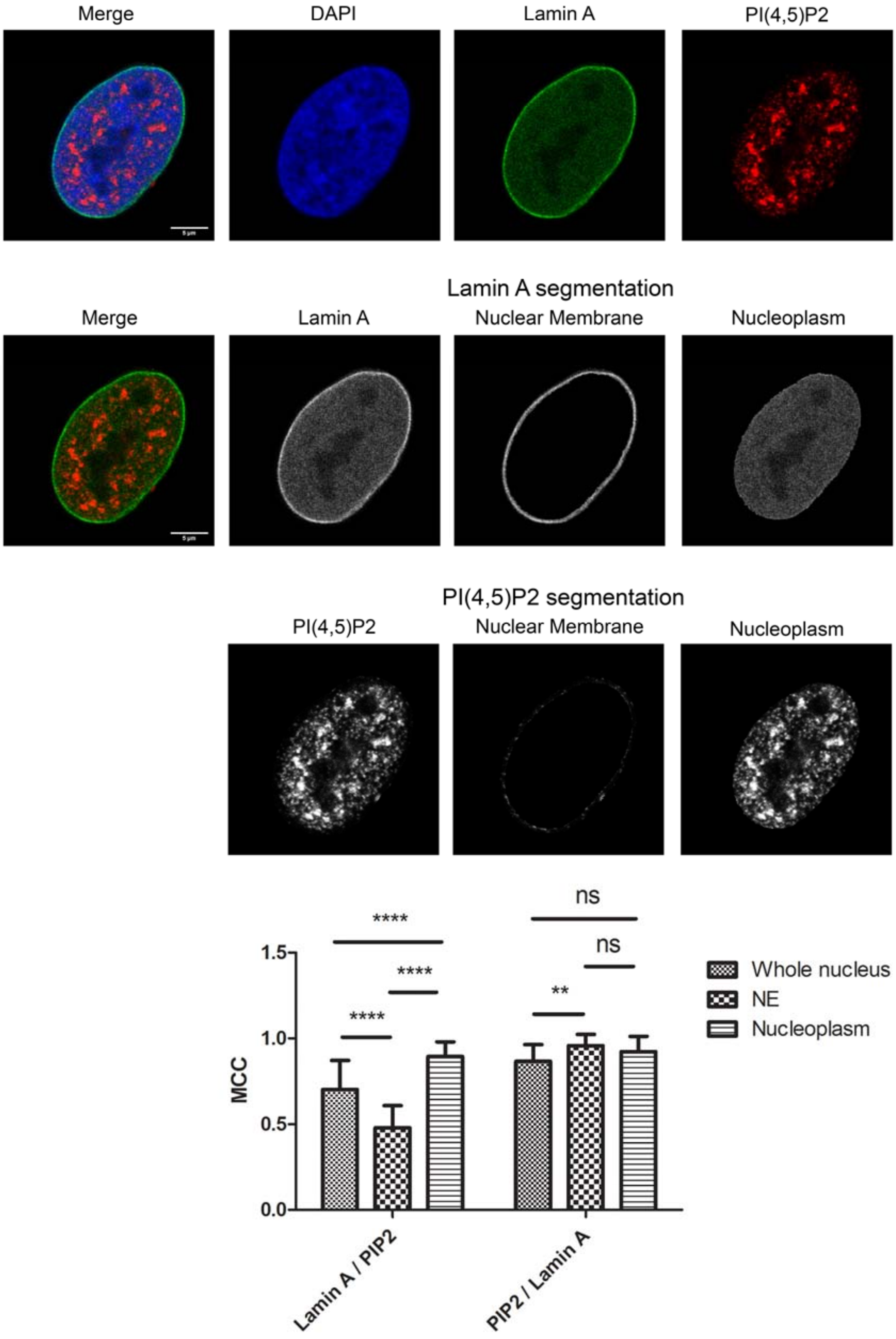

Supplementary Figure S5. Spatial localization of the lamin A/C–PI(4,5)P2 complex. Scanning confocal microscopy of U2OS cells transfected with WT–lamin A/C GFP tagged (green) and immunofluorescence labeled with PI(4,5)P2 antibody (red) and lamin. Images represent a middle optical section of the nucleus. Bar, 5 μm. Graph represents the statistical analysis of colocalization was performed on the whole area of the nuclear section acquired, as well as on the segmented pictures separately for lamina and the inner part of the nucleus (N=35). The MOC (Manders overlapping

coefficient) of lamin A/C over PI(4,5)P2, shown as the mean ± s.d., demonstrates a higher degree of colocalization between these two components in the nucleoplasmic segmentation ($p < 0.0001$). The MOC of PI(4,5)P2 over lamin A/C does not show statistically significant difference between NE and nucleoplasm. n.s.: non-significant; ** $p < 0.01$; **** $p < 0.0001$.
